# Endoscopic or No-Touch Vein Harvesting for CABG: What is Best for the Patient?

**DOI:** 10.5935/1678-9741.20160091

**Published:** 2016

**Authors:** Tomislav Kopjar, Stjepan Ivankovic, Melchior Luiz Lima, Bruno Botelho Pinheiro, Michael Richard Dashwood

**Affiliations:** 1 Department of Cardiac Surgery, University Hospital Centre Zagreb, Zagreb, Croatia.; 2 Department of Cardiovascular Surgery, Meridional Hospital, Vitoria, ES, Brazil.; 3 Department of Cardiovascular Surgery, Clinicord, Anis Rassi Hospital, Goiania, GO, Brazil.; 4 Surgical and Interventional Sciences, Royal Free Hospital Campus, University College Medical School, London, United Kingdom.

**Table:** 

**Abbreviations, acronyms & symbols**	
CABG	=Coronary artery bypass surgery
EACTS	=European Association for Cardio-Thoracic Surgery
ESC	=European Society of Cardiology
EVH	=Endoscopic vein harvesting
ISMICS	=International Society of Minimally Invasive Cardiothoracic Surgery
ITA	=Internal thoracic artery
OVH	=Open vein harvesting
SV	=Saphenous vein

**Fig. 1:**
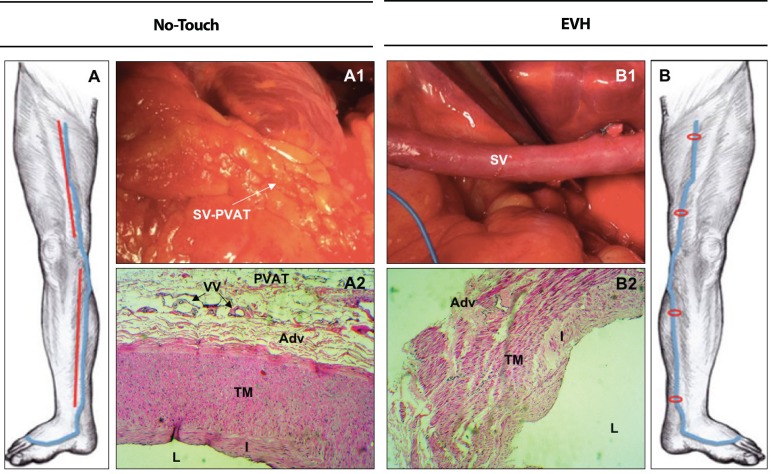
Examples of no-touch and endoscopic vein harvesting (EVH) techniques. **A –** Schematic drawing of the long incisions (red) at calf and thigh for exposure of saphenous vein (SV) (blue) by the no-touch technique. **B –** Schematic drawing of the small incisions (red) made at levels of upper thigh (~0.4 cm), knee (~3 cm) and ankle (~0.4 cm) using EVH system. **A1 –** Surgery photograph at the surface of the heart (right atrioventricular sulcus) showing the SV graft stripped of perivascular adipose tissue (PVAT) remaining intact when removed from the leg by no-touch technique. **B1 –** Surgery photograph at the surface of the heart (right atrioventricular sulcus) showing the SV graft stripped of PVAT when removed from the leg by EVH system. **A2 –** Histological photograph of a SV segment, harvested by the no-touch technique, with preserved PVAT. The histology shows an intact endothelium lining intima (I) of the lumen (L) and normal appearance of the tunica media (TM), adventitia (Adv), vasa vasorum (VV) and perivascular adipose tissue (PVAT). **B2 –** Histological photograph of a SV segment, harvested by the EVH technique, with total removal of PVAT. The histology shows regions of endothelial denudation and damage to the intima (I), tunica media (TM) and adventitial (Adv).

The saphenous vein (SV) is the most commonly used conduit for coronary artery bypass surgery (CABG)^[^^1^^]^, with minimally invasive endoscopic vein harvesting (EVH) being used in the majority of CABG in the USA^[^^2^^]^. While the benefits of EVH include reduced wound complications and improved cosmetic outcome, an inferior patency rate of EVH-SVs compared to those harvested by open vein harvesting (OVH) has been reported^[^^3^^]^ (Figure 1). Previous guidance in the United Kingdom advised that EVH should only be used with special arrangements^[^^4^^]^. This decision was based on data from the Project of Ex-vivo Vein Graft Engineering via Transfection (PREVENT) IV trial, where EVH-SV grafts showed higher failure rates than OVH grafts and, at 3 years, a higher death rate, myocardial infarction or revascularization compared to OVH grafts^[^^5^^]^. Originally, the PREVENT IV trial was designed as a randomized controlled trial to assess the effectiveness and safety of edifoligide on angiographic SV graft failure 12-18 months following CABG, as well as the effect of edifoligide on major adverse cardiac events throughout 5 years after CABG. Considering that the study by Lopes et al.^[^^5^^]^ is a secondary analysis, the results should be evaluated carefully and potential biases inherent to a non-randomized study design should be considered. However, data published subsequently included more patients and, although it was judged that "EVH did not show increased occlusion rates or incidences of reintervention, myocardial infarction or death for endoscopically harvested grafts...the Committee noted the importance of training and regular experience for any clinician doing this procedure" (United Kingdom NICE interventional procedure guidance 494, 2014)^[^^4^^]^. Simultaneously, the 2014 Guideline on Myocardial Revascularization of the European Society of Cardiology (ESC) and the European Association for Cardio-Thoracic Surgery (EACTS) acknowledged the lack of unequivocal evidence concerning patency rates of EVH-SVs and no inferiority in clinical outcomes associated with EVH^[^^6^^]^.

The unfavorable results regarding EVH-SV grafts are due to vascular damage inflicted where they are subjected to vascular trauma, including traction, adventitial stripping, and venous compression. Rousou et al.^[^^7^^]^ showed endothelial damage in EVH-SV grafts and demonstrated that cellular metabolic activity, viability and membrane damage to the endothelium is less in OVH compared with EVH grafts. Also, using optical coherence tomography, Kiani & Poston^[^^8^^]^ described marked damage to the adventitia of EVH-SV grafts as well as regions of endothelial denudation and abnormalities within the intima and deeper vessel layers, damage likely to affect graft patency.

At the time that EVH was described, an atraumatic, no-touch technique of harvesting the SV was introduced that, for up to 16 years, provides a superior graft with a patency rate comparable to the internal thoracic artery (ITA)^[^^9^^,^^10^^]^. This data is supported by a recent randomized trial from the Swedish group, which compared no-touch SV patency to the radial artery, providing further evidence for the superiority of no-touch SV grafts^[^^11^^]^. This is important given the general consensus amongst cardiac surgeons that radial artery patency is superior and therefore the second conduit of choice. While the incidence of wound complications in patients receiving no-touch SV is higher than in those receiving EVH grafts, they are similar to patients receiving OVH grafts and their performance is far superior, at ~90% *vs.* 50% long-term. Several studies have reported SV graft failure rates of up to 10% to 20% after 1 year and an additional 5% failure rate for each subsequent year with conventional OVH^[^^3^^,^^12^^-^^14^^]^. When performing OVH, the recent 2014 ESC/EACTS Guidelines on myocardial revascularization support the use of the notouch technique to reduce graft injury and improve patency^[^^6^^]^. There is evidence that the improved patency of no-touch SV over conventional OVH grafts is associated with a reduction in vascular damage^[^^15^^]^, maintaining normal vessel architecture, preservation of an intact endothelium^[^^16^^,^^17^^]^ and endotheliumderived NO^[^^17^^]^, as well as preservation of the *vasa vasorum*^[^^18^^,^^19^^]^ and the SVs surrounding cushion of perivascular fat^[^^20^^,^^21^^]^. The damage inflicted to the SV using both OVH and EVH affects both short- and long-term graft performance. For example, reduced luminal nitric oxide availability, due to endothelial injury, leads to increased platelet aggregation, thrombus formation and early graft occlusion^[^^12^^]^. The results of the immunohistochemical analysis with CD34, iNOS and three isoforms of nitric oxide synthase in the human SV removed conventionally showed an evident impairment of the endothelial function^[^^22^^-^^24^^]^. Damage to the outer layers of the SV, in particular the adventitial *vasa vasorum*, reduces transmural blood flow, resulting in medial ischemia, conditions that have been shown to promote neointimal hyperplasia and atherosclerosis, thereby affecting mid- and long-term graft patency^[^^19^^]^. The cushion of perivascular fat that remains intact using no-touch technique is an important source of adipocyte-derived vasodilator factors, suggested to play an important role in preventing venospasm at harvesting and post implantation^[^^20^^]^. In addition, this pronounced outermost layer of fat prevents kinking of excessively long grafts and provides mechanical support that protects the vein once it is subjected to arterial hemodynamics at completion of distal anastomoses and removal of arterial clamps^[^^18^^-^^21^^]^.

Considering the dramatic improvement reported for notouch SV grafts, it is surprising that this technique is limited to a few cardiac centers, mainly in Sweden and Brazil and, at a rough estimate, amount to less than 1000 cases per year worldwide. The reasons for this low take up rate are unclear, but may be associated with senior surgeons' resistance to change and/or unwillingness to "retrain", rather surprising given the rapid adoption of EVH to a present level of over 80% of all CABG in the USA^[^^3^^]^. EVH was recommended (Class I, Level A) to reduce wound-related complications, improve patient satisfaction, and decrease postoperative pain, hospital length of stay, and outpatient wound-management resources when compared with OVH^[^^25^^]^. While wound complications are reduced using EVH compared with OVH and no-touch-harvested SVs, graft performance of EVH grafts may be inferior^[^^25^^]^. Early no-touch SV harvest site complications have been reported to range from 11% to 18%^[^^25^^]^, considerably higher when compared to EVH, although similar to conventional OVH^[^^25^^]^. Furthermore, the learning curve for EVH is longer^[^^3^^]^, and although it has been suggested that EVH is a cost effective method for harvesting the SV, the 2005 Consensus Statement of the International Society of Minimally Invasive Cardiothoracic Surgery (ISMICS) concluded there was inadequate cost-effectiveness data to allow recommendations on the resource implications of OVH vs. EVH techniques^[^^25^^]^. Several recommendation statements and guidelines have emphasized the importance of experience when performing EVH^[^^4^^,^^6^^]^. Interestingly, although EVH may reduce wound complications, the recent study by Te Kolste et al.^[^^26^^]^ describes aspects of acute compartment syndrome, a rare, but serious, limb-threatening condition that may occur after CABG, especially following EVH. It is noteworthy that, over 10 years ago in their excellent review, Shuhaiber et al.^[^^27^^]^ stated "In the operating room, tissue manipulation and the role of the surgeon or surgical assistant is quite essential. The no-touch technique of handling tissues during harvesting should be adopted in order to preserve the endothelial integrity and function."

EVH is becoming popular in patients undergoing CABG in Brazil, a situation most likely influenced by the low wound healing complications and improved cosmetic outcome associated with this technique, in addition to its favored use in the USA. When considering the extra cost and problems regarding EVH-SV graft performance as described above, should the patient be made aware of the potential benefits of no-touch SV grafts? Should the benefits of no-touch SV harvesting be discussed and should the patient be given a choice?

**Table:** 

**Authors' roles & responsibilities**	
TK	Manuscript writing or critical review of its content; final manuscript approval
SI	Manuscript writing or critical review of its content; final manuscript approval
MLL	Manuscript writing or critical review of its content; final manuscript approval
BBP	Manuscript writing or critical review of its content; final manuscript approval
MRD	Manuscript writing or critical review of its content; final manuscript approval
